# Online-Delivered Group and Personal Exercise Programs to Support Low Active Older Adults’ Mental Health During the COVID-19 Pandemic: Randomized Controlled Trial

**DOI:** 10.2196/30709

**Published:** 2021-07-30

**Authors:** Mark R Beauchamp, Ryan M Hulteen, Geralyn R Ruissen, Yan Liu, Ryan E Rhodes, Colin M Wierts, Katrina J Waldhauser, Samantha H Harden, Eli Puterman

**Affiliations:** 1 School of Kinesiology University of British Columbia Vancouver, BC Canada; 2 School of Kinesiology Louisiana State University Baton Rouge, LA United States; 3 School of Exercise Science, Physical and Health Education University of Victoria Victoria, BC Canada; 4 Department of Human Nutrition, Foods, and Exercise Virginia Tech Blacksburg, VA United States

**Keywords:** COVID-19, randomized trial, mental health, physical activity

## Abstract

**Background:**

In response to the COVID-19 pandemic, experts in mental health science emphasized the importance of developing and evaluating approaches to support and maintain the mental health of older adults.

**Objective:**

The aim of this study was to assess whether a group-based exercise program relative to a personal exercise program (both delivered online) and waitlist control (WLC) can improve the psychological health of previously low active older adults during the early stages of the COVID-19 pandemic.

**Methods:**

The Seniors COVID-19 Pandemic and Exercise (SCOPE) trial was a 3-arm, parallel randomized controlled trial conducted between May and September 2020 in which low active older adults (aged ≥65 years) were recruited via media outlets and social media. After baseline assessments, consented participants were randomized to one of two 12-week exercise programs (delivered online by older adult instructors) or a WLC condition. A total of 241 older adults (n=187 women) provided baseline measures (via online questionnaires), were randomized (n_group_=80, n_personal_=82, n_control_=79), and completed measures every 2 weeks for the duration of the trial. The trial’s primary outcome was psychological flourishing. Secondary outcomes included global measures of mental and physical health, life satisfaction, and depression symptoms.

**Results:**

The results of latent growth modeling revealed no intervention effects for flourishing, life satisfaction, or depression symptoms (*P*>.05 for all). Participants in the group condition displayed improved mental health relative to WLC participants over the first 10 weeks (effect size [ES]=0.288-0.601), and although the week 12 effect (ES=0.375) was in the same direction the difference was not statistically significant (*P*=.089). Participants in the personal condition displayed improved mental health, when compared with WLC participants, in the same medium ES range (ES=0.293-0.565) over the first 8 weeks, and while the effects were of a similar magnitude at weeks 10 (ES=0.455, *P*=.069) and 12 (ES=0.258, *P*=.353), they were not statistically significant. In addition, participants in the group condition displayed improvements in physical health when compared with the WLC (ES=0.079-0.496) across all 12 weeks of the study following baseline. No differences were observed between the personal exercise condition and WLC for physical health (slope *P*=.271).

**Conclusions:**

There were no intervention effects for the trial’s primary outcome (ie, psychological flourishing). It is possible that the high levels of psychological flourishing at baseline may have limited the extent to which those indicators could continue to improve further through intervention (ie, potential ceiling effects). However, the intervention effects for mental and physical health point to the potential capacity of low-cost and scalable at-home programs to support the mental and physical health of previously inactive adults in the COVID-19 pandemic.

**Trial Registration:**

ClinicalTrials.gov NCT04412343; https://clinicaltrials.gov/ct2/show/NCT04412343

## Introduction

As the full scale and impact of the COVID-19 pandemic became evident in early 2020, older adults were identified as being particularly susceptible to severe illness and mortality [1]. National and local governments across the globe subsequently implemented a range of physical distancing mandates, which meant that older adults, in particular, were identified as being at risk of social isolation [2,3]. In direct response, mental health experts emphasized the importance of developing approaches to support and maintain the physical and mental health of older adults during this unprecedented time [2].

One widely scalable, nonpharmacological, and cost-effective approach promoted by the World Health Organization to support mental health during the pandemic corresponds to regular physical activity [4]. Although some correlational studies [5,6], including those focused on older adults [7,8], point to the possibility that regular physical activity may protect against depleted psychological well-being during the pandemic, there has been a distinct absence of experimental studies through which causality might be better ascertained. In this study we sought to examine the efficacy of 2 different types of exercise programs, both delivered online, to support the mental health of previously low active older adults (accumulating ≤60 minutes of moderate-intensity activity per week) within the context of the current COVID-19 pandemic in comparison to participants in a waitlist control (WLC) condition.

Results of previous (pre-COVID-19) experimental research suggest that when older adults exercise in groups with other older adults, led by older adult instructors, and have the opportunity to socially connect after classes, they displayed improvements in group cohesion (ie, they feel more connected) [9], adherence behavior [10], and psychological flourishing [11] when compared with older adults who exercise in classes with middle-aged and younger adults. Other research similarly indicates that when people exercise in group settings, especially within groups that are cohesive, they tend to stick with those programs to a greater extent than when exercising on their own [12]. Given the inability to exercise in community or social settings (eg, fitness/community centers) early in the COVID-19 pandemic, we sought to examine whether a virtually delivered group-based exercise program that sought to promote social connectedness among older adults would derive improved psychological well-being when compared with a personal exercise program (also delivered online) that did not operationalize social connectivity, as well as a WLC condition. The group-based program was informed by the tenets of self-categorization theory [13-16], which indicates that when people share common characteristics (eg, shared identity as older adults) and feel more connected to other group members, they will be more likely to retain their membership of those groups and display greater sense of well-being [17]. The results of a recent meta-analysis of interventions that were designed to foster a sense of social connectivity (and shared social identities) resulted in significant improvements in physical health as well as improvements (in the medium to large effect size [ES] range) in quality of life and cognitive health, as well as reductions in anxiety, depression, and stress [18]. In this trial, we identified psychological flourishing as the a priori primary outcome measure. Psychological flourishing has been identified as an important broad indicator of well-being [19,20], which involves feeling engaged in daily life, optimistic, having a sense of meaning and purpose, and having positive relationships [21]. Flourishing has also been identified as a viable target for intervention [22]. We hypothesized that older adults randomized to the virtual group program would display better well-being (higher levels of psychological flourishing) than those in a personal exercise condition, who in turn would display better well-being than WLCs. As secondary outcomes, we also assessed global measures of mental and physical health, life satisfaction, and depression symptoms. Furthermore, because individuals who live alone may benefit more from a group-based exercise program that fosters social connectivity compared with those who live with others, we investigated whether intervention effects are stronger in those who live alone versus with others. The above hypotheses were pre-registered via the Open Science Framework and ClinicalTrials.gov (see the “Methods” section).

## Methods

### Trial Design

The Seniors COVID-19 Pandemic and Exercise (SCOPE) study was a prospective, 3-arm, parallel, randomized controlled trial. The corresponding groups underwent a synchronous group-based exercise or an asynchronous personal exercise program (both delivered online), or a WLC condition. The study procedures were approved by the Research Ethics Board of The University of British Columbia, with the design, conduct, and reporting of this study adhering to the Consolidated Standards of Reporting Trials (CONSORT) guidelines [23] (Multimedia Appendix 1). The trial was pre-registered at ClinicalTrials.gov (#NCT04412343) and the Open Science Framework [24].

### Participants

Low active older adults (aged ≥65 years) without any medical contraindication that might prevent them from participating in moderate-intensity physical activity were eligible to participate. Additional inclusion criteria included (1) the ability to speak and read English, (2) currently live in Canada, (3) 1 participant in the study per household, and (4) able to access the internet at home via a personal smartphone, tablet (eg, iPad), or computer (with camera functionality). Activity status was assessed using the Stanford Leisure-Time Categorical Activity Item Version 2.2 (L-CAT; Version 2.2) [25], whereby participants select 1 of 6 descriptive categories ranging from inactive to very active. Consistent with previous use of the L-CAT to screen for low active participants [26], only participants who scored between 1 and 3 were eligible to participate. Item 1 corresponds to activity “no more than once or twice a month,” item 2 reflects undertaking “light activities once or twice a week,” and item 3 reflects “moderate-intensity activities 3 times per week for 15-20 minutes each time or sport or moderate-intensity activities once per week for 45-60 minutes.” As such, item 3 (≤60 minutes of moderate-intensity activity per week) reflects a threshold below current recommendations of 150 minutes of moderate-intensity activity per week for older adults [27]. Prescreening also involved completion of the Physical Activity Readiness Questionnaire for Everyone (PARQ+) and the Electronic Physical Activity Readiness Medical Examination (ePARmed-X+ [28]). If the ePAR-medX+ highlighted that physician approval was required prior to joining the program, the respective individual was informed that this approval was required before they could enroll in the study. Following the initial screening process, informed consent was obtained.

Participants were recruited via social media advertisements (eg, Twitter, Facebook) and news coverage related to the trial (radio, print media), which directed them to the study website. Interested participants were invited to contact the trial coordinator (RH) who scheduled a scripted eligibility screening phone call with a member of the research team. After ascertaining eligibility, interested participants provided informed consent and completed baseline measures for all study measures (ie, demographics plus all health measures) online via Qualtrics (Freedom of Information and Protection of Privacy Act [FIPPA] compliant [29]).

### Study Interventions

Participants in the 2 experimental conditions were subsequently directed to a password-protected and secured web platform housed by the first author’s institution (ie, Canvas). This platform provided access to the appropriate exercise programs and intervention materials. Individuals randomized to undergo the group-based exercise program received an adapted version of the group program that was previously implemented for older adults for in-person exercise classes [10]. Specifically, participants had the opportunity to take part in group exercise classes delivered via an online communications platform (ie, Zoom housed within Canvas) by older adult exercise instructors (n_men_=3, n_women_=4; mean age 68.29 [SD 8.90] years) that were employed at a local community center and had considerable experience delivering older adult exercise classes. Classes were offered 7 days a week at 9 am Pacific Standard Time (12 noon Eastern Standard Time, 1 pm Atlantic Standard Time), and lasted approximately 50-60 minutes. Classes included a warm-up component, moderate-intensity exercises as the core component of the class, and a cool-down period, and were designed specifically for older adults to include strength, flexibility, balance, and aerobic components. Consistent with international guidelines for weekly physical activity by older adults (150 minutes of moderate-to-vigorous intensity physical activity [27]), participants were encouraged to attend at least three classes each week. Classes were hosted on Zoom by a trained research assistant (RH, GR, and CW) who provided technical assistance to ensure that the classes were accessible to participants.

Instructors were provided with autonomy support [30], whereby they could choose the exercises included in each class; however, to ensure sufficient support, instructors were directed to ensure that all exercises could be completed in the home environment with minimal need for equipment (resistance bands were sent, by postal mail, to all participants to facilitate strength-based exercises). Instructors were encouraged to use language in their classes to foster a sense of “us” and that “we’re in this together” (ie, to develop a sense of social identity and connectedness even though classes were delivered virtually). At the end of classes, participants had the opportunity to connect in small groups (via Zoom breakout groups) to socially connect over a beverage (coffee, water) from their own homes. If participants missed the live class, they could access a recording of that class in their own time. Participants were also sent, by postal mail, a program t-shirt to foster a sense of distinctiveness [31].

The same older adult instructors that delivered the group exercise classes also delivered the personal exercise classes (they were blind to the trial hypotheses). Classes in the personal exercise condition were matched for frequency, duration, intensity, and content with the group exercise classes but, in this instance, instructors used language during the classes that referred to themselves as each participant’s personal trainer/coach, and language directed to the individual and not any group. That is, no sense of “groupness” or “shared social identity/connectivity” was primed. Classes were pre-recorded and accessed via Canvas, which meant that they could be accessed any time in the day of the participants’ choosing. Participants in this condition did not have the opportunity to interact with other program participants after classes had ended, and did not receive the same program t-shirts designed to foster a sense of group distinctiveness.

Older adults randomized to the control condition were asked to go about their daily lives for the duration of the 12-week trial. They were asked to complete the same questionnaires (and were remunerated in the same way) as those in the other 2 conditions. At the end of the 12-week trial, participants in this condition were provided with access to (and supports associated with) the personal exercise program described above.

Participants in all 3 conditions were sent questionnaires related to the trial’s measures (see below) for completion (via Qualtrics) at the end of weeks 2, 4, 6, 8, 10, and 12. In return for survey completion, at each time point, participants were provided Can $10 (US $8) (Can $70 [US $56] total; baseline plus 6 follow-up assessments). Participants also received up to Can $50 (US $40) if any costs were incurred for obtaining medical clearance from their respective family doctor.

### Measures

The primary outcome measure was psychological flourishing, which we assessed using Diener and colleagues’ [21] 8-item measure. Exemplar items include “I lead a purposeful and meaningful life” and “I am engaged and interested in my daily activities,” with all items anchored on a 7-point Likert scale from “strongly disagree” (1) to “strongly agree” (7). Responses to the flourishing scale demonstrated acceptable reliability, with Cronbach α values ranging from .90 to .94 across the 7 time points. Secondary outcomes included global measures of mental and physical health, life satisfaction, and depression symptoms. Global mental and physical health were assessed using separate 1-item measures developed by Hays and colleagues [32]. Specifically, mental health was assessed using the item “In general, would you say your MENTAL OR EMOTIONAL HEALTH is excellent, very good, good, fair, or poor?,” while physical health was assessed using the item “In general, would you say your PHYSICAL HEALTH is excellent, very good, good, fair, or poor?,” with each item anchored from “poor” (1) to “excellent” (5). Life satisfaction was assessed using the 1-item question by Fleeson [33] that asked participants “Using a scale from 0 to 10 where 0 means ‘the worst possible life overall’ and 10 means ‘the best possible life overall’, how would you rate your life overall these days?.” Depression symptoms were assessed using the 10-item Center for Epidemiologic Studies Depression Scale (CES-D) [34] that asked participants to report the frequency of depression symptoms over the past week. Exemplar items include “I felt that everything I did was an effort” and “I felt depressed,” with all items anchored by “Rarely or none of the time (less than 1 day)” and “Most or all of the time (5-7 days).” Responses to the CES-D demonstrated acceptable reliability, with Cronbach α values ranging from .83 to .87 across the 7 time points. In addition, participants completed measures of chronic health conditions [35], as well as demographic measures that recorded sex and gender, age, type of dwelling, ethnicity, sexual orientation, smoking behavior, height, weight, education level, household income level, employment status, marital status, and living situation (ie, living alone versus with others). Participant engagement in each of the physical activity programs, as a measure of program adherence, was operationalized via the data analytics for each individual within the Canvas platform, where each of the classes/sessions were provided. As a manipulation check, participants were considered to have attended a class/session if they recorded 10 or more minutes of class/session access.

### Sample Size Calculation

To account for interdependence among observations (ie, multiple observations within the same participant), we conducted the power analysis using Optimal Design Software [36]. On the basis of 7 observations (baseline, plus weeks 2, 4, 6, 8, 10, and 12), a total sample size of 527 was identified as necessary to detect a small ES (in psychological flourishing) of δ=0.25, with intraclass correlation coefficient set at 0.05, power (1–β) at 80%, and α at .05 with 7 time points. To account for a study attrition rate of 10% (over the course of the study), a sample size of 600 was considered sufficient to examine the latent growth models (LGMs) proposed in this trial.

### Randomization and Blinding

Participants were stratified to ensure equal distribution of men and women across conditions. Sequence generation was completed separately for men and women using the Research Randomizer [37] tool for researchers, with blocks of 3 unique numbers (1, 2, and 3) that designated 1 of the 3 randomization groups. A researcher external to the project team generated the sequence and remained blind to participant allocation. Participants were randomized in the order they completed baseline surveys. Although the trial coordinator (RH) was aware of condition assignment (following randomization), there were no experimenter or investigator expectancy effects related to the mental health outcome measures as all assessments occurred online (ie, online questionnaires). Once baseline measures were completed, the trial coordinator contacted each participant to inform him/her of the condition assignment as a result of the trial’s randomization procedures.

### Changes to the Trial

On July 17, 2020, study recruitment was terminated for several reasons. First, during our recruitment window (June and July 2020), there were some major global events (eg, protests, riots, and political events) which limited our ability to get the word out via the media. Although we had some success with media recruitment (national radio, TV, print media), our recruitment did not have the anticipated reach. Second, we wanted to keep the recruitment window similar across participants: we anticipated that the experiences of older adults early in the pandemic would be notably different to those experienced months later (eg, summer versus winter, along with geopolitical changes across time). Closing new enrollments at that point meant that all participants commenced the study at the same time of the year (within a 5-week window) and had started at approximately the same time as one another in relation to the unfolding pandemic. Third, although a sample of 600 was required to detect a small effect, under the same parameters as originally presented (ie, intraclass correlation coefficient set at 0.05, power [1–β] at 80%, and α at .05 with 7 time points including baseline, weeks 2, 4, 6, 8, 10, and 12), a sample of 209 was required to detect a small-to-medium effect of δ=0.40 and a sample of 134 was required to detect a medium-sized effect of δ=0.50. Thus, even after accounting for the original attrition rate of 10%, a sample of 241 was deemed sufficient to detect small-to-medium and medium ESs of 0.40 and 0.50, respectively. As there were no feasibility/efficacy data to sufficiently gauge the size of an intervention effect in the context of a pandemic, we felt it was appropriate to cease new enrollments, while acknowledging that the trial would not be sufficiently powered to detect small effects, but would be well powered to detect small-to-medium and medium-sized effects. More information on these changes can be accessed here [38].

We originally also sought to examine whether any intervention effects might be more pronounced among those with lower mental health at baseline [24]. Unfortunately, we were precluded from conducting these subgroup analyses due to the resultant small sample size and instability of parameter estimates. For example, research using the CES-D has identified a threshold score of 10 or more as indicative of depression symptomology [34]. In our study, 76 older adults met this criterion on the basis of their baseline scores.

### Statistical Analyses

We conducted our main data analyses for the 5 outcome variables using latent growth modeling based on a structural equation modeling framework, including all randomized participants (intention-to-treat analyses), using the Mplus version 7.4 software [39] with maximum likelihood robust estimation (Multimedia Appendix 2). As the data were collected on multiple occasions over 12 weeks following baseline assessments, we tested both linear and nonlinear trends. First, we conducted an unconditional growth model, and compared linear (Multimedia Appendix 3) and quadratic (Multimedia Appendix 4) growth models, and determined the optimal model through commonly used model fit indices. This corresponded to the comparative fit index (CFI), the root mean square error of approximation (RMSEA), and the standardized root mean square residual (SRMR). The criteria for evaluating model fit was designated with CFI values over 0.90, and RMSEA and SRMR values less than 0.08 [40,41]. Quadratic models were utilized to take account of nonlinear growth trends. Second, to test the hypothesized treatment effects, we included the intervention conditions (personal exercise versus group exercise) in the analysis, and controlled for the effects of covariates, including sex, age, living situation (ie, “alone” versus “with others”), and chronic health conditions. In light of our a priori hypothesis that living status would moderate the intervention effects, we included the interaction of living situation and experimental conditions in this step. We computed ESs at each time point using Feingold’s approach [42-44] for growth modeling (equivalent to Cohen *d*).

## Results

### Overview

**Figure 1:**
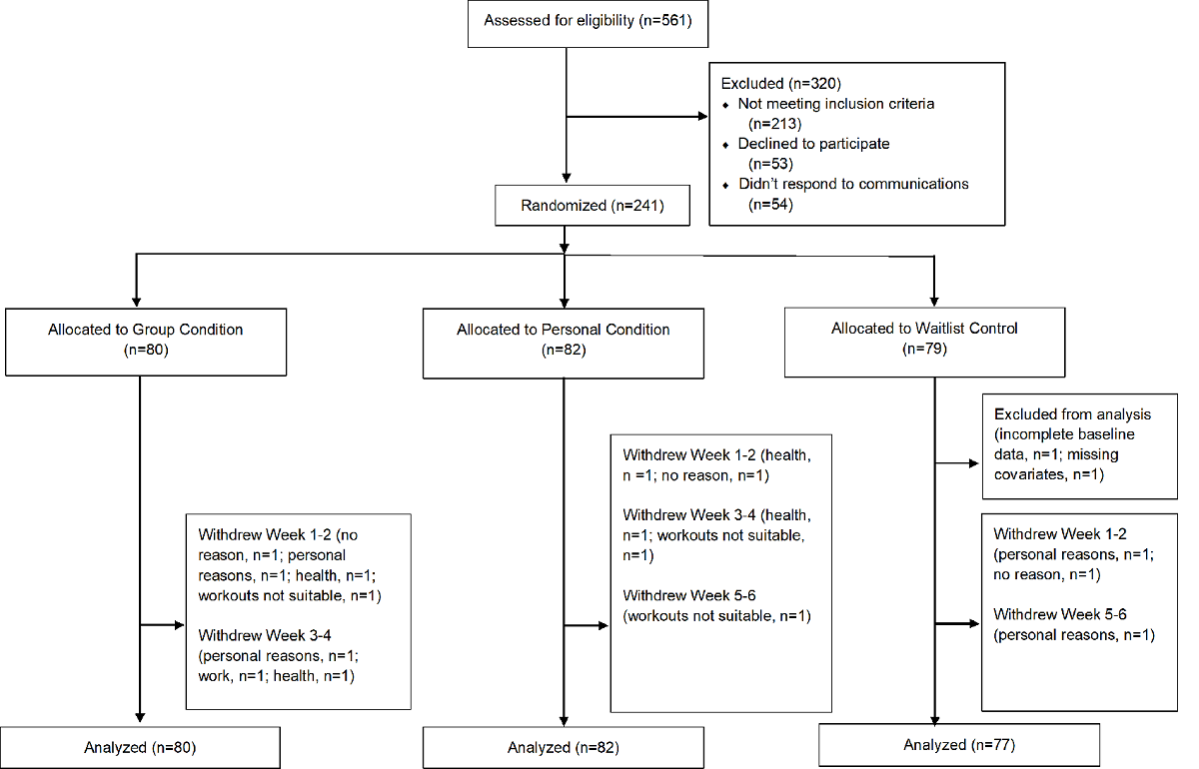
CONSORT flow diagram.

**Figure 2:**
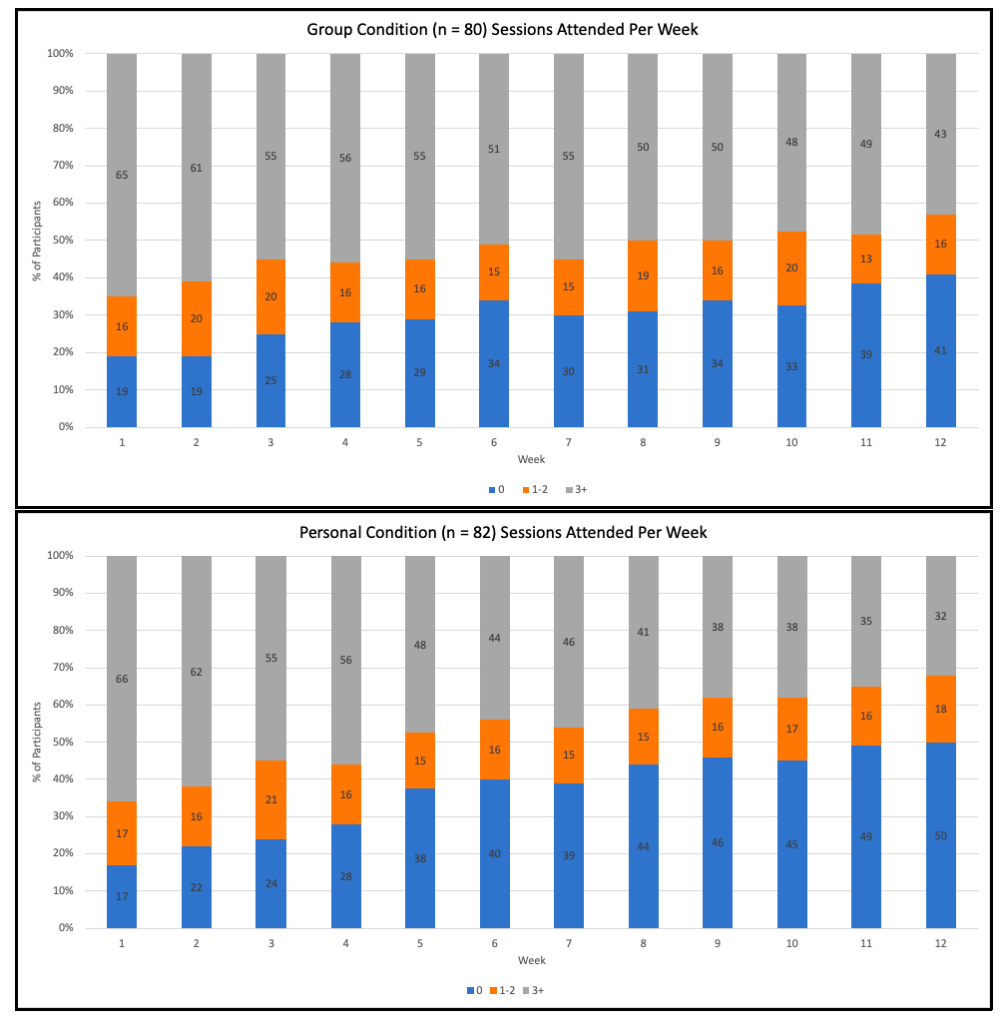
Program attendance in the two experimental conditions across the 12-week trial.

Five hundred and sixty-one individuals were screened and, based on eligibility, 241 adults aged 65-94 (mean age 73.03 [SD 5.42] years) enrolled between May 23 and July 12, 2020 (Figure 1). Descriptive statistics for the sociodemographic factors are presented in Multimedia Appendix 5. There were no differences between groups at baseline (as indicated by the nonsignificant intercepts in Multimedia Appendices 6-10) with regard to any of the 5 dependent measures assessed in the study. Correlations among the study variables at each time point are presented in Multimedia Appendices 11-17. Exercise session attendance for the 2 experimental conditions across the 12 weeks of the trial is presented in Figure 2.

### Intervention Groups Versus Control Condition on Study Outcomes

#### Flourishing

**Figure 3:**
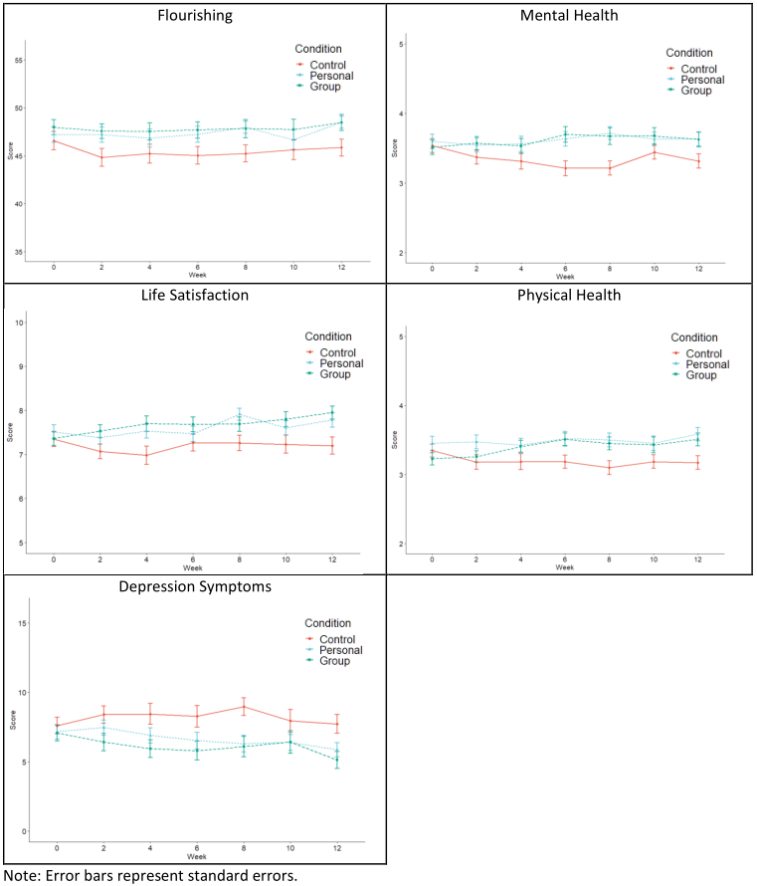
Trajectories for mental health outcomes over the course of the trial.

An LGM that accounted for quadratic change displayed good model fit (CFI=0.967, RMSEA=0.048, SRMR=0.067) for flourishing (Multimedia Appendix 6). There were no differences between the 2 intervention conditions and control condition at baseline (as denoted by nonsignificant intercepts; Multimedia Appendix 6 and Figure 3). Older adults living with others versus alone (intercept estimate=3.562, SE=1.171, *P*=.030, 95% CI 0.197-6.926) and those with fewer chronic health conditions (intercept estimate=–0.977, SE=0.205, *P*<.001, 95% CI –1.379 to –0.576) displayed higher levels of flourishing at baseline. After controlling for covariates, there was no significant intervention effect for either personal or group exercise conditions in comparison to the WLC and a nonsignificant intervention condition by living situation interaction (as denoted by nonsignificant slopes; Multimedia Appendix 6).

#### Mental Health

An LGM that accounted for quadratic change displayed good model fit (CFI=0.992, RMSEA=0.025, SRMR=0.031) for mental health (Multimedia Appendix 7). There were no differences between the 2 intervention conditions and control condition at baseline (as denoted by nonsignificant intercepts; Multimedia Appendix 7). With regard to covariates, men reported better mental health at baseline (intercept estimate=0.286, SE=0.132, *P*=.030, 95% CI 0.027-0.544), as did those who were older (intercept estimate=0.044, SE=0.010, *P*<.001, 95% CI 0.024-0.064), and those with fewer chronic health conditions (intercept estimate=–0.116, SE=0.027, *P*<.001, 95% CI –0.169 to –0.064). After controlling for covariates, both the personal exercise (slope estimate=0.291, SE=0.123, *P*=.010, 95% CI 0.050-5.32) and group exercise (slope estimate=0.282, SE=0.099, *P*=.004, 95% CI 0.088-0.476) conditions displayed improved mental health when compared with the WLC condition (Multimedia Appendix 7 and Figure 3). The significant quadratic effects for both intervention conditions in relation to the WLC illustrate differences in curvature of the slopes when compared with the control condition (Multimedia Appendix 7 and Figure 3). The differences in mental health between the personal exercise condition and the WLC were in the medium ES range (ES=0.293-0.565) over the first 8 weeks and although the effects were of a similar magnitude at weeks 10 (ES=0.455, *P*=.069) and 12 (ES=0.258, *P*=.353), they were not statistically significant. The differences in mental health between the group condition and WLC were in the same medium ES range over the first 10 weeks (ES=0.288-0.601), and although the week 12 effect (ES=0.375) was in the same direction, the difference was not statistically significant (*P*=.089). The intervention condition by living situation interactions were nonsignificant (Multimedia Appendix 7).

#### Physical Health

An LGM that accounted for linear change displayed good model fit (CFI=0.977, RMSEA=0.037, SRMR=0.037) for self-reported physical health (Multimedia Appendix 8). Model fit did not improve by modeling quadratic change. With regard to covariates, participants who were older (intercept estimate=0.033, SE=0.009, *P*<.001, 95% CI 0.015-0.050), and those with fewer chronic health conditions (intercept estimate=–0.116, SE=0.025, *P*<.001, 95% CI –0.168 to –0.071) reported better physical health at baseline. After controlling for covariates, participants in the group condition displayed improvements in physical health when compared with the WLC (slope estimate=0.063, SE=0.030, *P*<.001, 95% CI 0.004-0.121), with the ESs observed in the small to medium range (ES=0.079-0.496) across all 12 weeks of the study following baseline (Multimedia Appendix 8 and Figure 3). After controlling for covariates, the difference between the personal exercise condition and WLC was not significant. None of the condition by living situation interactions were significant (Multimedia Appendix 8).

#### Life Satisfaction

An LGM that accounted for linear change displayed good model fit (CFI=0.947, RMSEA=0.059, SRMR=0.067) for life satisfaction (Multimedia Appendix 9). Model fit did not improve by modeling quadratic change. With regard to covariates, men reported greater life satisfaction at baseline (intercept estimate=0.623, SE=0.191, *P*=.001, 95% CI 0.250-0.997), as did those who were older (intercept estimate=0.039, SE=0.014, *P*=.007, 95% CI 0.011-0.068) and those with fewer chronic health conditions (intercept estimate=–0.177, SE=0.039, *P*<.001, 95% CI –0.252 to –0.101). After controlling for covariates, there was no significant intervention effect for either personal or group exercise condition in comparison to the WLC and the condition by living situation interactions were also nonsignificant (as denoted by nonsignificant slopes; Multimedia Appendix 9 and Figure 3).

#### Depression Symptoms

An LGM that accounted for quadratic change displayed good model fit (CFI=0.992, RMSEA=0.024, SRMR=0.024) for depression symptoms (Multimedia Appendix 10). With regard to covariates, women reported less depression symptoms at baseline (intercept estimate=–1.454, SE=0.639, *P*=.023, 95% CI –2.707 to –0.202), as did those who were older (intercept estimate=–0.133, SE=0.050, *P*=.008, 95% CI –0.232 to –0.035]), while those with more chronic health conditions reported higher depression symptoms (intercept estimate=0.703, SE=0.150, *P*<.001, 95% CI 0.408-0.997) at baseline (Multimedia Appendix 10). There were no significant intervention effects and none of the condition by living status interactions were significant (Multimedia Appendix 10 and Figure 3).

## Discussion

### Principal Findings

The overall purpose of this study was to test the efficacy of 2 physical activity programs to support previously low active older adults’ psychological and physical well-being early in the COVID-19 pandemic. Both physical activity interventions were delivered online, with one designed to foster a sense of social connectivity, and the other designed to support independent physical activity, and compared against a control condition. After displaying comparable levels of program adherence over the first 4 weeks of the trial, participants in the group program displayed improved adherence compared with those in the personal exercise program; over the last 4 weeks the proportion of participants attending 3 or more sessions per week was 10% or more in the group condition than in the personal condition (Figure 1). Despite this, there were no intervention effects for either condition, in relation to the trial’s primary outcome, psychological flourishing, or measures of life satisfaction and depression symptoms. Both intervention conditions did, however, display significant intervention effects (in the medium ES range) for a global/omnibus measure of mental health when compared with the control condition. In addition, participants in the group exercise condition demonstrated significant intervention effects, again in the medium ES range, for self-reported physical health when compared with controls.

Early in the pandemic, older adults were identified as being particularly at risk of isolation and depleted well-being [2,3], and as such represented the focus of intervention in this trial. As the first few months of the pandemic progressed, the results of large-scale epidemiology studies in North America revealed that, perhaps contrary to initial expectations, older adults displayed the lowest prevalence of psychological distress within any age group (adults aged 18-29 displayed the highest levels of distress) [45]. With this in mind, it is notable that older adults who were screened for eligibility and enrolled in the study displayed generally good psychological health at baseline (ie, high mean levels of flourishing and low mean levels of depression symptoms). Indeed, it is likely that the high levels of psychological flourishing reported for the overall sample at baseline may have limited the extent to which those indicators could continue to improve further through intervention (ie, potential ceiling effects). It is also noteworthy that psychological flourishing represents a multicomponent indicator of well-being [22] that includes aspects such as having positive relationships, feelings of competence, and having meaning and purpose in life [21], and so it is certainly conceivable that the interventions tested in this study were not sufficiently potent to improve such a broad multicomponent indicator. The same could also be said of life satisfaction, which also displayed null effects in this trial.

Nevertheless, the trial did result in significant intervention effects for both experimental conditions in relation to global indicators of mental health when compared with the control condition. In addition, it is noteworthy that involvement in the group condition resulted in medium-sized effects in self-reported physical health. While the trial resulted in intervention effects for these 2 measures, and null effects for the other 3 mental health measures (flourishing, life satisfaction, and depression symptoms), it is notable that the trajectories, or patterns, of all 5 of the study measures (Figure 3) are directly comparable to one another (with depression symptoms displaying mirrored trajectories; with lower depression symptoms reflecting better psychological health). We also examined whether living situation might moderate the effects of the interventions in relation to the targeted study outcomes, and hypothesized a priori that the physical activity programs (in particular, the group-based program) would be more effective in contributing to participant well-being for those who live alone than with others. No significant condition by living situation interactions were observed, which suggests that no differential intervention effects occurred based on participants’ living status.

From a knowledge translation perspective several findings are worthy of note. First, the 2 intervention conditions were directly matched in relation to the content and frequency of classes/sessions, with the same older adult instructors delivering the classes across conditions. Although the personal exercise condition had built-in flexibility, whereby participants could access the classes/sessions during times of their own choosing, the adherence data indicate that the opportunity to exercise alongside other older adults (in an online group-based program) provided an added draw to sustain their involvement. Indeed, while the adherence levels were directly comparable across conditions for the first 4 weeks of the trial, over the remaining 8 weeks (likely as participants become more familiar with one another) those in the group program displayed improved adherence behaviors. Although older adults may not accrue the same quality of connections with other class members that occurs within more typical in-person groups, the adherence data indicated that online groups can act to substantively retain older adults’ involvement in physical activity programs (at least in the context of a global pandemic). Second, the programs delivered in this trial were designed in such a way that all exercises could be completed in the home environment with minimal need for equipment. Thus, provided that participants had access to the internet at home via a personal smartphone, tablet (eg, iPad), or computer, there were no barriers to participation. We recognize that some older adults face digital inequalities that limit their access to the internet (and programs delivered via the internet) [46]. Nevertheless, with the proportion of older adults who have access to the internet doubling between 2007 and 2016 in Canada (from 32% to 68%) [47], and with trends expected to improve further [47], programs such as those delivered in this trial have considerable potential to be delivered to older adults, either in circumstances such as the current COVID-19 pandemic, or in other contexts such as living in remote or rural communities. Such online programs also have considerable potential to be delivered at scale.

Despite the contributions of the study, limitations must be acknowledged. The most substantive limitation corresponds to the deviation from the initial target sample size (as described in the “Methods” section). Although we utilized social media (Facebook, Twitter) to recruit participants, we found that the overwhelming majority of participants, most likely due to the nature of the older adult demographic, were alerted to the study via news reports in the national/provincial press. We had good uptake from a handful of news reports/stories (to facilitate recruitment), but were unable to secure our targeted sample size. Nevertheless, our eventual sample size (n=241) may well have precluded us from detecting significant between-group effects within the LGMs (smaller sample sizes tend to produce increased standard errors, thus reducing statistical power sensitivity to detect between-group differences). The second limitation corresponded to our measure of physical health. Because of the physical distancing recommendations that existed at the time of conducting the study, it was not possible to conduct in-person assessments of physical health. As such, we utilized a self-report global measure of physical health, which could at best be described as a proxy for actual physical health. As a final limitation, we originally planned to examine whether the effects would be more pronounced for those who displayed worsened mental health at baseline; however, we were precluded from conducting this subgroup analyses due to the small number of participants who displayed identifiably depleted levels of mental health at baseline (eg, CES-D scores ≥10 [34]). Nevertheless, despite these limitations, the study represents one of the few randomized trials to evaluate the efficacy of physical activity interventions during the COVID-19 pandemic, with the findings revealing that virtually delivered interventions are feasible and, when delivered in a group setting, can aid the retention of previously low active older adults. In addition, the results provided some indication that both physical activity programs showed improvements in mental health when compared with control participants, which represents a notable outcome for older adults in the current COVID-19 pandemic.

### Conclusions

In response to calls from mental health experts [2] early in the COVID-19 pandemic to design and implement programs to support the mental and physical health of older adults, we sought to examine the efficacy of 2 interventions through the implementation of a pre-registered randomized controlled trial. Both interventions were delivered online, to support previously low active older adults engaging in physical activity while ensuring that they maintained physical distancing (as part of prevailing government pandemic-related public health mandates). Although no significant intervention effects resulted in relation to the trial’s primary outcome (ie, psychological flourishing), the intervention effects for both the group and personal conditions in relation to mental and physical health (in the medium ES range) point to the capacity of low-cost and scalable at-home programs, delivered online, to support older adults’ well-being in the COVID-19 pandemic, as well as other remote or hard-to-reach rural settings.

## Appendix

Multimedia Appendix 1 CONSORT-eHEALTH checklist (V 1.6.1).

Multimedia Appendix 2 Mplus code.

Multimedia Appendix 3 Latent growth modeling path diagram (modeling linear change) for explanatory variables in relation to the putative dependent measure over the 12-week trial.

Multimedia Appendix 4 Latent growth modeling path diagram (modeling quadratic change) for explanatory variables in relation to the putative dependent measure over the 12-week trial.

Multimedia Appendix 5 Participant demographic information.

Multimedia Appendix 6 Latent growth model (accounting for quadratic change) for psychological flourishing.

Multimedia Appendix 7 Latent growth model (accounting for quadratic change) for mental health.

Multimedia Appendix 8 Latent growth model (accounting for linear change) for physical health.

Multimedia Appendix 9 Latent growth model (accounting for linear change) for life satisfaction.

Multimedia Appendix 10 Quadratic latent growth model for depressive symptomology.

Multimedia Appendix 11 Baseline correlations among study variables.

Multimedia Appendix 12 Correlations among study variables at week 2.

Multimedia Appendix 13 Correlations among study variables at week 4.

Multimedia Appendix 14 Correlations among study variables at week 6.

Multimedia Appendix 15 Correlations among study variables at week 8.

Multimedia Appendix 16 Correlations among study variables at week 10.

Multimedia Appendix 17 Correlations among study variables at week 12.

## Abbreviations

CES-D: Center for Epidemiologic Studies Depression Scale
CFI: comparative fit index
ePARmed-X+: Electronic Physical Activity Readiness Medical Examination
ES: effect size
FIPPA: Freedom of Information and Protection of Privacy Act
L-CAT: Stanford Leisure-Time Categorical Activity Item
LGM: latent growth model
PARQ+: Physical Activity Readiness Questionnaire for Everyone
RMSEA: root mean square error of approximation
SCOPE: Seniors COVID-19 Pandemic and Exercise
SRMR: standardized root mean square residual
WLC: waitlist control
