# Supplementary material for: Online-Delivered Group and Personal Exercise Programs to Support Low Active Older Adults’ Mental Health During the COVID-19 Pandemic: Randomized Controlled Trial
Source: J Med Internet Res. 2021 Jul 30;23(7):e30709. doi: 10.2196/30709 (PMC8330630; doi:10.2196/30709)
Supplement: Multimedia Appendix 2 [file jmir_v23i7e30709_app2.docx]

**Multimedia Appendix 2. Mplus code.**

**Mplus Code (Linear Latent Growth Models):**

USEVARIABLES ARE g1 g2 man age living chrotot

phyh0 phyh1 phyh2 phyh3 phyh4 phyh5 phyh6

lvxg1 lvxg2;

DEFINE:

lvxg1 = living*g1;

lvxg2 = living*g2;

ANALYSIS: ESTIMATOR = MLR;

STITERATIONS=4000;

ITERATION = 20000;

!PARAMETERIZATION = THETA;

MODEL:

i s | phyh0@0 phyh1@1 phyh2@2 phyh3@3 phyh4@4 phyh5@5 phyh6@6;

i s ON g1 g2 living lvxg1 lvxg2 man age chrotot;

i(v0);

s ON g1 (bs1);

s ON g2 (bs2);

phyh0 - phyh6(r0 -r6);

MODEL CONSTRAINT:

NEW(g1dt1 g1dt2 g1dt3 g1dt4 g1dt5 g1dt6);

g1dt1 = (bs1*1)/sqrt(v0+r0/2+r1/2);

g1dt2 = (bs1*2 )/sqrt(v0+r0/3+r1/3+r2/3);

g1dt3 = (bs1*3)/sqrt(v0+r0/4+r1/4+r2/4+r3/4);

g1dt4 = (bs1*4)/sqrt(v0+r0/5+r1/5+r2/5+r3/5+r4/5);

g1dt5 = (bs1*5)/sqrt(v0+r0/6+r1/6+r2/6+r3/5 +r4/6+r5/6);

g1dt6 = (bs1*6)/sqrt(v0+r0/7+r1/7+r2/7+r3/7 +r4/7+r5/7+r6/7);

NEW(g2dt1 g2dt2 g2dt3 g2dt4 g2dt5 g2dt6);

g2dt1 = (bs2*1)/sqrt(v0+r0/2+r1/2);

g2dt2 = (bs2*2)/sqrt(v0+r0/3+r1/3+r2/3);

g2dt3 = (bs2*3)/sqrt(v0+r0/4+r1/4+r2/4+r3/4);

g2dt4 = (bs2*4)/sqrt(v0+r0/5+r1/5+r2/5+r3/5+r4/5);

g2dt5 = (bs2*5)/sqrt(v0+r0/6+r1/6+r2/6+r3/5+r4/6+r5/6);

g2dt6 = (bs2*6)/sqrt(v0+r0/7+r1/7+r2/7+r3/7+r4/7+r5/7+r6/7);

OUTPUT: SAMPSTAT CINTERVAL STANDARDIZED RESIDUAL MODINDICES (3.84);

**Mplus Code (Quadratic Latent Growth Models):**

USEVARIABLES ARE g1 g2 man age living chrotot

mental0 mental1 mental2 mental3 mental4 mental5 mental6

lvxg1 lvxg2;

DEFINE:

lvxg1 = living*g1;

lvxg2 = living*g2;

ANALYSIS: ESTIMATOR = ML;

STITERATIONS=5000;

ITERATION = 20000;

!PARAMETERIZATION = THETA;

MODEL:

i s q | mental0@0 mental1@1 mental2@2 mental3@3 mental4@4 mental5@5 mental6@6;

i s q ON g1 g2 living lvxg1 lvxg2 man age chrotot;

i(v0);

s ON g1 (bs1);

s ON g2 (bs2);

q ON g1 (bq1);

q ON g2 (bq2);

mental0 - mental6 (r0 -r6);

MODEL CONSTRAINT:

NEW(g1dt1 g1dt2 g1dt3 g1dt4 g1dt5 g1dt6);

g1dt1 = (bs1*1 + bq1*1)/sqrt(v0+r0/2+r1/2);

g1dt2 = (bs1*2 + bq1*4)/sqrt(v0+r0/3+r1/3+r2/3);

g1dt3 = (bs1*3 + bq1*9)/sqrt(v0+r0/4+r1/4+r2/4+r3/4);

g1dt4 = (bs1*4 + bq1*16)/sqrt(v0+r0/5+r1/5+r2/5+r3/5+r4/5);

g1dt5 = (bs1*5 + bq1*25)/sqrt(v0+r0/6+r1/6+r2/6+r3/5 +r4/6+r5/6);

g1dt6 = (bs1*6 + bq1*36)/sqrt(v0+r0/7+r1/7+r2/7+r3/7 +r4/7+r5/7+r6/7);

NEW(g2dt1 g2dt2 g2dt3 g2dt4 g2dt5 g2dt6);

g2dt1 = (bs2*1 + bq2*1)/sqrt(v0+r0/2+r1/2);

g2dt2 = (bs2*2 + bq2*4)/sqrt(v0+r0/3+r1/3+r2/3);

g2dt3 = (bs2*3 + bq2*9)/sqrt(v0+r0/4+r1/4+r2/4+r3/4);

g2dt4 = (bs2*4 + bq2*16)/sqrt(v0+r0/5+r1/5+r2/5+r3/5+r4/5);

g2dt5 = (bs2*5 + bq2*25)/sqrt(v0+r0/6+r1/6+r2/6+r3/5+r4/6+r5/6);

g2dt6 = (bs2*6 + bq2*36)/sqrt(v0+r0/7+r1/7+r2/7+r3/7+r4/7+r5/7+r6/7);

OUTPUT: SAMPSTAT CINTERVAL STANDARDIZED RESIDUAL MODINDICES (3.84);
