# Supplementary material for: Online-Delivered Group and Personal Exercise Programs to Support Low Active Older Adults’ Mental Health During the COVID-19 Pandemic: Randomized Controlled Trial
Source: J Med Internet Res. 2021 Jul 30;23(7):e30709. doi: 10.2196/30709 (PMC8330630; doi:10.2196/30709)

**Multimedia Appendix 4. Latent growth modeling path diagram (modeling quadratic change) for explanatory variables in relation to the putative dependent measure over the 12-week trial.**


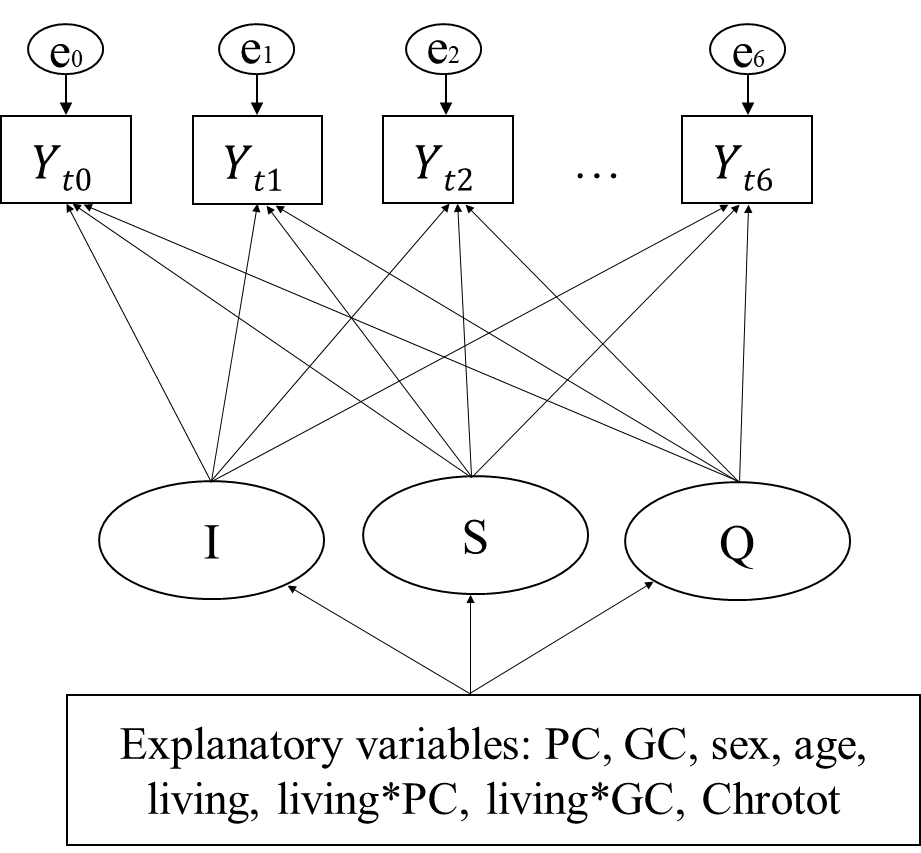

Supplement: Multimedia Appendix 4 [file jmir_v23i7e30709_app4.docx]
