# Supplementary material for: Online-Delivered Group and Personal Exercise Programs to Support Low Active Older Adults’ Mental Health During the COVID-19 Pandemic: Randomized Controlled Trial
Source: J Med Internet Res. 2021 Jul 30;23(7):e30709. doi: 10.2196/30709 (PMC8330630; doi:10.2196/30709)
Supplement: Multimedia Appendix 5 [file jmir_v23i7e30709_app5.docx]

**Multimedia Appendix 5. Participant demographic information.**

|  | Group (n = 80) | Personal (n = 82) | Control (n = 79) | Total (n = 241) |
| --- | --- | --- | --- | --- |
| Sex n (%) |  |  |  |  |
| *Male* | 15 (18.8) | 18 (22.0) | 20 (25.3) | 53 (22.0) |
| *Female* | 65 (81.3) | 64 (78.0) | 59 (76.7) | 188 (88.0) |
| *Age, M (SD), years* | 73.0 (5.0) | 74.1 (6.2) | 72.0 (4.8) | 73.03 (5.42) |
| *Height, M (SD), cm* | 164.3 (11.2) | 165.6 (9.7) | 164.3 (11.9) | 164.6 (10.9) |
| *Missing n (%)* | 0 (0.0) | 2 (2.4) | 6 (7.6) | 8 (3.3) |
| *Weight, M (SD), kg* | 74.9 (15.6) | 73.2 (15.1) | 76.0 (18.6) | 74.7 (16.4) |
| *Missing n (%)* | 0 (0.0) | 0 (0.0) | 3 (3.8) | 3 (1.2) |
| Indigenous n (%) |  |  |  |  |
| *Yes* | 0 (0) | 0 (0) | 1 (1.3) | 1 (0.4) |
| *No* | 80 (100) | 82 (100) | 77 (97.5) | 239 (99.2) |
| *Missing* | 0 (0) | 0 (0) | 1 (1.3) | 1 (0.4) |
| Disability n (%) |  |  |  |  |
| *Yes* | 3 (3.8) | 4 (4.9) | 1 (1.3) | 8 (3.3) |
| *No* | 75 (93.8) | 78 (95.1) | 77 (97.5) | 230 (95.4) |
| *Prefer not to answer* | 2 (2.5) | 0 (0) | 0 (0) | 2 (0.8) |
| *Missing* | 0 (0) | 0 (0) | 1 (1.3) | 1 (0.4) |
| Member of a visible minority n (%) |  |  |  |  |
| *Yes* | 13 (16.3) | 5 (6.1) | 8 (10.1) | 26 (10.8) |
| *No* | 65 (81.3) | 76 (92.7) | 69 (87.3) | 210 (87.1) |
| *Prefer not to answer* | 2 (2.5) | 1 (1.2) | 1 (1.3) | 4 (1.7) |
| *Missing* | 0 (0) | 0 (0) | 1 (1.3) | 1 (0.4) |
| LGBTQ n (%) |  |  |  |  |
| *Yes* | 3 (3.8) | 0 (0) | 2 (2.5) | 5 (2.1) |
| *No* | 76 (95.0) | 81 (98.8) | 76 (96.2) | 233 (96.7) |
| *Prefer not to answer* | 1 (1.3) | 1 (1.2) | 0 (0) | 2 (0.8) |
| *Missing* | 0 (0) | 0 (0.0) | 1 (1.3) | 1 (0.4) |
| Highest level of education n (%) |  |  |  |  |
| Less than a high school diploma | 1 (1.3) | 0 (0) | 0 (0) | 1 (0.4) |
| High school diploma or equivalent | 8 (10.0) | 10 (12.2) | 7 (8.9) | 25 (10.4) |
| Trade certificate/diploma | 2 (2.5) | 3 (3.7) | 3 (3.8) | 8 (3.3) |
| College or other non-university certificate (other than trades) | 12 (15.0) | 10 (12.2) | 13 (16.5) | 35 (14.5) |
| University certificate/diploma below a Bachelor’s | 9 (11.3) | 7 (8.5) | 4 (5.1) | 20 (8.3) |
| Bachelor’s Degree | 17 (21.3) | 22 (26.8) | 26 (32.9) | 65 (27.0) |
| Higher than a Bachelors Degree | 30 (37.5) | 29 (35.4) | 24 (30.4) | 83 (34.4) |
| Prefer not to answer | 1 (1.3) | 1 (1.2) | 1 (1.3) | 3 (1.2) |
| Missing | 0 (0) | 0 (0) | 1 (1.3) | 1 (0.4) |
| Dwelling type n (%) |  |  |  |  |
| Single-detached home | 34 (42.5) | 50 (61.0) | 37 (46.8) | 121 (50.2) |
| Apartment building (5 storeys or less) | 13 (16.3) | 8 (9.8) | 11 (13.9) | 32 (13.3) |
| Apartment building (5 storeys or more) | 18 (22.5) | 13 (15.9) | 13 (16.5) | 44 (18.3) |
| Row or town house | 10 (12.5) | 8 (9.8) | 10 (12.7) | 28 (11.6) |
| Semi-detached | 2 (2.5) | 1 (1.2) | 3 (3.8) | 6 (2.5) |
| Apartment/flat in a duplex | 1 (1.3) | 0 (0) | 3 (3.8) | 4 (1.7) |
| Other single attached home | 0 (0) | 1 (1.2) | 1 (1.3) | (2 (0.8) |
| Movable dwelling | 1 (1.3) | 1 (1.2) | 0 (0) | 2 (0.8) |
| Missing | 1 (1.3) | 0 (0) | 1 (1.3) | 2 (0.8) |
| Current living situation n (%) |  |  |  |  |
| Living alone | 38 (47.5) | 22 (26.8) | 34 (43.0) | 94 (39.0) |
| Living with others | 42 (52.5) | 60 (73.2) | 44 (55.7) | 146 (60.6) |
| Missing | 0 (0) | 0 (0) | 1 (1.3) | 1 (0.4) |
| Household income n (%) |  |  |  |  |
| $0-20,000 | 2 (2.5) | 2 (2.4) | 2 (2.5) | 6 (2.5) |
| $20,001-40,000 | 9 (11.2) | 3 (3.7) | 8 (10.2) | 20 (8.3) |
| $40,001-60,000 | 9 (11.2) | 11 (13.4) | 10 (12.6) | 30 (12.4) |
| $60,001-80,000 | 8 (10.0) | 12 (14.6) | 14 (17.8) | 34 (14.1) |
| $80,001-100,000 | 10 (12.5) | 9 (11.0) | 10 (12.7) | 29 (12.0) |
| $100,001-120,000 | 6 (7.5) | 8 (9.8) | 5 (6.3) | 19 (7.9) |
| $120,001-140,000 | 7 (8.8) | 6 (7.3) | 4 (5.0) | 17 (7.1) |
| $140,001-160,000 | 4 (5.0) | 5 (6.1) | 4 (5.0) | 13 (5.4) |
| $160,001 or above | 5 (6.3) | 10 (12.2) | 8 (10.2) | 23 (9.5) |
| Do not know/prefer not to answer | 20 (25.0) | 15 (18.3) | 13 (16.4) | 48 (20.0) |
| Missing | 0 (0) | 1 (1.2) | 1 (1.3) | 2 (0.8) |
