# Supplementary material for: Online-Delivered Group and Personal Exercise Programs to Support Low Active Older Adults’ Mental Health During the COVID-19 Pandemic: Randomized Controlled Trial
Source: J Med Internet Res. 2021 Jul 30;23(7):e30709. doi: 10.2196/30709 (PMC8330630; doi:10.2196/30709)
Supplement: Multimedia Appendix 6 [file jmir_v23i7e30709_app6.docx]

**Multimedia Appendix 6. Latent growth model (accounting for quadratic change) for psychological flourishing.**

|  | **Variables** | **Estimates** | **SE** | ***p-value*** | **95% CI** |
| --- | --- | --- | --- | --- | --- |
| Intercept | Personal Condition | 2.328 | 1.927 | 0.227 | -1.450, 6.105 |
|  | Group Condition | 2.710 | 1.673 | 0.105 | -0.568, 5.988 |
|  | Living Situation | 3.562 | 1.717 | **0.038** | **0.197, 6.926** |
|  | Living Situation X Personal Condition | -2.458 | 2.334 | 0.292 | -7.033, 2.118 |
|  | Living Situation X Group Condition | -0.790 | 2.119 | 0.709 | -4.943, 3.362 |
|  | Gender | 1.011 | 0.986 | 0.306 | -0.922, 2.944 |
|  | Age | 0.109 | 0.076 | 0.149 | -0.039, 0.258 |
|  | Chronic Conditions | -0.977 | 0.205 | **<0.001** | **-1.379, -0.576** |
|  |  |  |  |  |  |
| Slope | Personal Condition | -0.239 | 0.963 | 0.804 | -2.126, 1.648 |
|  | Group Condition | -0.003 | 0.781 | 0.997 | -1.534, 1.528 |
|  | Living Situation | -0.210 | 0.797 | 0.792 | -1.771, 1.352 |
|  | Living Situation X Personal Condition | 0.886 | 1.145 | 0.439 | -1.359, 3.131 |
|  | Living Situation X Group Condition | 0.356 | 1.024 | 0.728 | -1.650, 2.362 |
|  | Gender | -0.043 | 0.552 | 0.938 | -1.124, 1.038 |
|  | Age | 0.001 | 0.036 | 0.969 | -0.070, 0.073 |
|  | Chronic Conditions | 0.166 | 0.106 | 0.117 | -0.042, 0.374 |
|  |  |  |  |  |  |
| Quadratic Function | Personal Condition | 0.054 | 0.137 | 0.695 | -0.215, 0.322 |
|  | Group Condition | 0.018 | 0.112 | 0.875 | -0.201, 0.236 |
|  | Living Situation | 0.002 | 0.114 | 0.983 | -0.220, 0.225 |
|  | Living Situation X Personal Condition | -0.122 | 0.162 | 0.454 | -0.439, 0.196 |
|  | Living Situation X Group Condition | -0.065 | 0.148 | 0.658 | -0.355, 0.224 |
|  | Gender | -0.014 | 0.078 | 0.863 | -0.167, 0.140 |
|  | Age | -0.004 | 0.006 | 0.432 | -0.015, 0.006 |
|  | Chronic Conditions | -0.024 | 0.015 | 0.097 | -0.053, 0.004 |

**Note:** Personal Condition = Personal exercise condition (anchored against control condition), Group Condition = Group exercise condition (anchored against control condition), Living Situation = Living with others (anchored against living alone), Gender = Male (anchored against referent Female, Chronic Conditions = Number of chronic health conditions.
