# Supplementary material for: Online-Delivered Group and Personal Exercise Programs to Support Low Active Older Adults’ Mental Health During the COVID-19 Pandemic: Randomized Controlled Trial
Source: J Med Internet Res. 2021 Jul 30;23(7):e30709. doi: 10.2196/30709 (PMC8330630; doi:10.2196/30709)
Supplement: Multimedia Appendix 7 [file jmir_v23i7e30709_app7.docx]

**Multimedia Appendix 7. Latent growth model (accounting for quadratic change) for mental health.**

|  | **Variables** | **Estimates** | **SE** | ***p-value*** | **95% CI** |
| --- | --- | --- | --- | --- | --- |
| Intercept | Personal Condition | -0.026 | 0.226 | 0.907 | -0.469, 0.416 |
|  | Group Condition | -0.154 | 0.193 | 0.425 | -0.533, 0.225 |
|  | Living Situation | 0.178 | 0.189 | 0.345 | -0.192, 0.549 |
|  | Living Situation X Personal Condition | -0.097 | 0.277 | 0.726 | -0.640, 0.446 |
|  | Living Situation X Group Condition | 0.205 | 0.262 | 0.433 | -0.308, 0.719 |
|  | Gender | 0.286 | 0.132 | **0.030** | **0.027, 0.544** |
|  | Age | 0.044 | 0.010 | **<0.001** | **0.024, 0.064** |
|  | Chronic Conditions | -0.116 | 0.027 | **<0.001** | **-0.169, -0.064** |
|  |  |  |  |  |  |
| Slope | Personal Condition | 0.291 | 0.123 | **0.018** | **0.050, 0.532** |
|  | Group Condition | 0.282 | 0.099 | **0.004** | **0.088, 0.476** |
|  | Living Situation | 0.035 | 0.096 | 0.712 | -0.152, 0.223 |
|  | Living Situation X Personal Condition | -0.090 | 0.148 | 0.541 | -0.379, 0.199 |
|  | Living Situation X Group Condition | -0.036 | 0.136 | 0.793 | -0.302, 0.231 |
|  | Gender | 0.026 | 0.070 | 0.715 | -0.112, 0.163 |
|  | Age | -0.015 | 0.005 | **0.004** | **-0.026, -0.005** |
|  | Chronic Conditions | 0.021 | 0.014 | 0.149 | -0.007, 0.049 |
|  |  |  |  |  |  |
| Quadratic Function | Personal Condition | -0.042 | 0.020 | **0.033** | **-0.081, -0.003** |
|  | Group Condition | -0.038 | 0.016 | **0.016** | **-0.069, -0.007** |
|  | Living Situation | -0.010 | 0.015 | 0.500 | -0.040, 0.020 |
|  | Living Situation X Personal Condition | 0.018 | 0.024 | 0.458 | -0.029, 0.064 |
|  | Living Situation X Group Condition | 0.005 | 0.022 | 0.833 | -0.038, 0.047 |
|  | Gender | -0.007 | 0.011 | 0.550 | -0.029, 0.015 |
|  | Age | 0.002 | 0.001 | **0.030** | **0.000, 0.004** |
|  | Chronic Conditions | -0.003 | 0.002 | 0.240 | -0.007, 0.002 |
|  |  |  |  |  |  |
| Effect Size | |  |  |  |  |
|  | Personal Condition T1 | 0.293 | 0.124 | **0.018** | **0.050, 0.536** |
|  | Personal Condition T2 | 0.476 | 0.201 | **0.018** | **0.082, 0.870** |
|  | Personal Condition T3 | 0.565 | 0.241 | **0.019** | **0.093, 1.038** |
|  | Personal Condition T4 | 0.563 | 0.254 | **0.026** | **0.066, 1.060** |
|  | Personal Condition T5 | 0.455 | 0.250 | 0.069 | -0.035, 0.945 |
|  | Personal Condition T6 | 0.258 | 0.277 | 0.353 | -0.286, 0.801 |
|  | Group Condition T1 | 0.288 | 0.100 | **0.004** | **0.091, 0.484** |
|  | Group Condition T2 | 0.476 | 0.163 | **0.003** | **0.157, 0.795** |
|  | Group Condition T3 | 0.580 | 0.196 | **0.003** | **0.196, 0.963** |
|  | Group Condition T4 | 0.601 | 0.206 | **0.003** | **0.198, 1.005** |
|  | Group Condition T5 | 0.528 | 0.202 | **0.009** | **0.131, 0.924** |
|  | Group Condition T6 | 0.375 | 0.221 | 0.089 | -0.057, 0.807 |

**Note:** Personal condition = Personal exercise condition (anchored against control condition), Group Condition = Group exercise condition (anchored against control condition), Living Situation = Living with others (anchored against living alone), Gender = Male (anchored against referent Female, Chronic Conditions = Number of chronic health conditions. Effect sizes represent differences between the two intervention conditions and the control condition at weeks 2 (time 1) to 12 (time 6) following randomization.
