# Supplementary material for: Online-Delivered Group and Personal Exercise Programs to Support Low Active Older Adults’ Mental Health During the COVID-19 Pandemic: Randomized Controlled Trial
Source: J Med Internet Res. 2021 Jul 30;23(7):e30709. doi: 10.2196/30709 (PMC8330630; doi:10.2196/30709)
Supplement: Multimedia Appendix 8 [file jmir_v23i7e30709_app8.docx]

**Multimedia Appendix 8. Latent growth model (accounting for linear change) for physical health.**

|  | **Variables** | **Estimates** | **SE** | ***p-value*** | **95% CI** |
| --- | --- | --- | --- | --- | --- |
| Intercept | Personal Condition | 0.225 | 0.198 | 0.256 | -0.163, 0.612 |
|  | Group Condition | 0.046 | 0.172 | 0.790 | -0.292, 0.383 |
|  | Living Situation | 0.278 | 0.186 | 0.136 | -0.088, 0.643 |
|  | Living Situation X Personal Condition | -0.242 | 0.250 | 0.333 | -0.733, 0.248 |
|  | Living Situation X Group Condition | -0.113 | 0.231 | 0.624 | -0.565, 0.339 |
|  | Gender | -0.130 | 0.125 | 0.301 | -0.376, 0.116 |
|  | Age | 0.033 | 0.009 | **<0.001** | **0.015, 0.050** |
|  | Chronic Conditions | -0.119 | 0.025 | **<0.001** | **-0.168, -0.071** |
|  |  |  |  |  |  |
| Slope | Personal Condition | 0.048 | 0.043 | 0.271 | -0.037, 0.113 |
|  | Group Condition | 0.063 | 0.030 | **0.035** | **0.004, 0.121** |
|  | Living Situation | -0.03 | 0.027 | 0.260 | -0.083, 0.022 |
|  | Living Situation X Personal Condition | -0.003 | 0.049 | 0.957 | -0.099, 0.094 |
|  | Living Situation X Group Condition | -0.001 | 0.037 | 0.987 | -0.074, 0.073 |
|  | Gender | 0.040 | 0.018 | **0.023** | **0.006, 0.075** |
|  | Age | -0.003 | 0.001 | 0.051 | **-0.006, 0.000** |
|  | Chronic Conditions | 0.006 | 0.004 | 0.123 | -0.001, 0.013 |
|  |  |  |  |  |  |
| Effect Sizes | |  |  |  |  |
|  | Personal Condition T1 | 0.060 | 0.055 | 0.274 | -0.048, 0.169 |
|  | Personal Condition T2 | 0.122 | 0.112 | 0.273 | -0.096, 0.341 |
|  | Personal Condition T3 | 0.186 | 0.17 | 0.274 | -0.147, 0.519 |
|  | Personal Condition T4 | 0.251 | 0.229 | 0.273 | -0.198, 0.700 |
|  | Personal Condition T5 | 0.312 | 0.284 | 0.273 | -0.245, 0.869 |
|  | Personal Condition T6 | 0.379 | 0.345 | 0.273 | -0.298, 1.056 |
|  | Group Condition T1 | 0.079 | 0.038 | **0.036** | **0.005, 0.153** |
|  | Group Condition T2 | 0.160 | 0.076 | **0.036** | **0.010, 0.310** |
|  | Group Condition T3 | 0.244 | 0.116 | **0.036** | **0.016, 0.471** |
|  | Group Condition T4 | 0.328 | 0.156 | **0.036** | **0.022, 0.635** |
|  | Group Condition T5 | 0.408 | 0.194 | **0.035** | **0.028, 0.789** |
|  | Group Condition T6 | 0.496 | 0.236 | **0.035** | **0.034, 0.958** |

**Note:** Personal Condition = Personal exercise condition (anchored against control condition), Group Condition = Group exercise condition (anchored against control condition), Living Situation = Living with others (anchored against living alone), Gender = Male (anchored against referent Female, Chronic Conditions = Number of chronic health conditions. Effect sizes represent differences between the two intervention conditions and the control condition at weeks 2 (time 1) to 12 (time 6) following randomization.
