# Supplementary material for: Online-Delivered Group and Personal Exercise Programs to Support Low Active Older Adults’ Mental Health During the COVID-19 Pandemic: Randomized Controlled Trial
Source: J Med Internet Res. 2021 Jul 30;23(7):e30709. doi: 10.2196/30709 (PMC8330630; doi:10.2196/30709)
Supplement: Multimedia Appendix 9 [file jmir_v23i7e30709_app9.docx]

**Multimedia Appendix 9. Latent growth model (accounting for linear change) for life satisfaction.**

|  | **Variables** | **Estimates** | **SE** | ***p-value*** | **95% CI** |
| --- | --- | --- | --- | --- | --- |
| Intercept | Personal Condition | 0.312 | 0.328 | 0.342 | -0.332, 0.956 |
|  | Group Condition | 0.279 | 0.277 | 0.313 | -0.263, 0.822 |
|  | Living Situation | 0.512 | 0.271 | 0.059 | -0.020, 1.044 |
|  | Living Situation X Personal Condition | -0.314 | 0.401 | 0.434 | -1.099, 0.472 |
|  | Living Situation X Group Condition | -0.048 | 0.376 | 0.898 | -0.785, 0.688 |
|  | Gender | 0.623 | 0.191 | **0.001** | **0.250, 0.997** |
|  | Age | 0.039 | 0.014 | **0.007** | **0.011, 0.068** |
|  | Chronic Conditions | -0.177 | 0.039 | **<0.001** | **-0.252, -0.101** |
|  |  |  |  |  |  |
| Slope | Personal Condition | 0.073 | 0.068 | 0.284 | -0.061, 0.207 |
|  | Group Condition | 0.057 | 0.054 | 0.291 | -0.049, 0.162 |
|  | Living Situation | -0.028 | 0.051 | 0.588 | -0.128, 0.073 |
|  | Living Situation X Personal Condition | -0.029 | 0.081 | 0.715 | -0.188, 0.129 |
|  | Living Situation X Group Condition | 0.006 | 0.073 | 0.938 | -0.138, 0.149 |
|  | Gender | -0.036 | 0.038 | 0.336 | -0.110, 0.037 |
|  | Age | -0.008 | 0.003 | **0.005** | **-0.014, -0.002** |
|  | Chronic Conditions | 0.009 | 0.008 | 0.266 | -0.007, 0.024 |

**Note:** Personal Condition = Personal exercise condition (anchored against control condition), Group Condition = Group exercise condition (anchored against control condition), Living Situation = Living with others (anchored against living alone), Gender = Male (anchored against referent Female, Chronic Conditions = Number of chronic health conditions.
