# Supplementary material for: Online-Delivered Group and Personal Exercise Programs to Support Low Active Older Adults’ Mental Health During the COVID-19 Pandemic: Randomized Controlled Trial
Source: J Med Internet Res. 2021 Jul 30;23(7):e30709. doi: 10.2196/30709 (PMC8330630; doi:10.2196/30709)
Supplement: Multimedia Appendix 10 [file jmir_v23i7e30709_app10.docx]

**Multimedia Appendix 10. Quadratic latent growth model for depressive symptomology.**

|  | **Variables** | **Estimates** | **SE** | ***p-value*** | **95% CI** |
| --- | --- | --- | --- | --- | --- |
| Intercept | Personal Condition | -0.610 | 1.344 | 0.650 | -3.243, 2.024 |
|  | Group Condition | -1.248 | 1.063 | 0.241 | -3.332, 0.836 |
|  | Living Situation | -2.897 | 1.136 | **0.011** | **-5.124, -0.669** |
|  | Living Situation X Personal Condition | 1.445 | 1.603 | 0.367 | -1.697, 4.587 |
|  | Living Situation X Group Condition | 0.897 | 1.464 | 0.540 | -1.972, 3.766 |
|  | Gender | -1.454 | 0.639 | **0.023** | **-2.707, -0.202** |
|  | Age | -0.133 | 0.050 | **0.008** | **-0.232, -0.035** |
|  | Chronic Conditions | 0.703 | 0.150 | **<0.001** | **0.408, 0.997** |
|  |  |  |  |  |  |
| Slope | Personal Condition | -0.669 | 0.764 | 0.381 | -2.167, 0.829 |
|  | Group Condition | -1.011 | 0.699 | 0.148 | -2.381, 0.358 |
|  | Living Situation | 0.364 | 0.672 | 0.588 | -0.953, 1.680 |
|  | Living Situation X Personal Condition | -0.295 | 0.856 | 0.730 | -1.972, 1.382 |
|  | Living Situation X Group Condition | -0.018 | 0.846 | 0.983 | -1.676, 1.639 |
|  | Gender | -0.318 | 0.343 | 0.354 | -0.990, 0.354 |
|  | Age | 0.030 | 0.029 | 0.307 | -0.027, 0.087 |
|  | Chronic Conditions | -0.058 | 0.078 | 0.459 | -0.210, 0.095 |
|  |  |  |  |  |  |
| Quadratic Function | Personal Condition | 0.061 | 0.109 | 0.576 | -0.153, 0.275 |
|  | Group Condition | 0.118 | 0.098 | 0.225 | -0.073, 0.310 |
|  | Living Situation | -0.019 | 0.096 | 0.843 | -0.206, 0.168 |
|  | Living Situation X Personal Condition | 0.053 | 0.124 | 0.669 | -0.190, 0.296 |
|  | Living Situation X Group Condition | 0.002 | 0.124 | 0.987 | -0.241, 0.245 |
|  | Gender | 0.052 | 0.051 | 0.305 | -0.047, 0.152 |
|  | Age | -0.001 | 0.004 | 0.748 | -0.010, 0.007 |
|  | Chronic Conditions | 0.013 | 0.012 | 0.263 | -0.010, 0.036 |

**Note:** Personal Condition = Personal exercise condition (anchored against control condition), Group Condition = Group exercise condition (anchored against control condition), Living Situation = Living with others (anchored against living alone), Gender = Male (anchored against referent Female, Chronic Conditions = Number of chronic health conditions.
