# Supplementary material for: Online-Delivered Group and Personal Exercise Programs to Support Low Active Older Adults’ Mental Health During the COVID-19 Pandemic: Randomized Controlled Trial
Source: J Med Internet Res. 2021 Jul 30;23(7):e30709. doi: 10.2196/30709 (PMC8330630; doi:10.2196/30709)
Supplement: Multimedia Appendix 11 [file jmir_v23i7e30709_app11.docx]

**Multimedia Appendix 11. Baseline correlations among study variables.**

| Variable | 1 | 2 | 3 | 4 | 5 | 6 | 7 | 8 |
| --- | --- | --- | --- | --- | --- | --- | --- | --- |
| 1. Gender |  |  |  |  |  |  |  |  |
| Sig (2-tailed) |  |  |  |  |  |  |  |  |
|  |  |  |  |  |  |  |  |  |
| 1. Age | .04 |  |  |  |  |  |  |  |
| Sig (2-tailed) | .55 |  |  |  |  |  |  |  |
|  |  |  |  |  |  |  |  |  |
| 1. Living Situation | .16^b^ | -.17^a^ |  |  |  |  |  |  |
| Sig (2-tailed) | .01 | .007 |  |  |  |  |  |  |
|  |  |  |  |  |  |  |  |  |
| 1. Chronic Conditions | -.20 ^a^ | -.02 | -.01 |  |  |  |  |  |
| Sig (2-tailed) | .002 | .81 | .89 |  |  |  |  |  |
|  |  |  |  |  |  |  |  |  |
| 1. Satisfaction with Life | .26 ^a^ | .17^a^ | .12 | -.35^a^ |  |  |  |  |
| Sig (2-tailed) | < .001 | .009 | .06 | < .001 |  |  |  |  |
|  |  |  |  |  |  |  |  |  |
| 1. Physical Health | -.00 | .22^a^ | .04 | -.31^a^ | .35^a^ |  |  |  |
| Sig (2-tailed) | .98 | < .001 | .52 | < .001 | < .001 |  |  |  |
|  |  |  |  |  |  |  |  |  |
| 1. Mental Health | .22 ^a^ | .22^a^ | .10 | -.29^a^ | .68^a^ | .44^a^ |  |  |
| Sig (2-tailed) | < .001 | < .001 | .13 | < .001 | < .001 | < .001 |  |  |
|  |  |  |  |  |  |  |  |  |
| 1. Flourishing | .08 | .04 | .15^b^ | -.28^a^ | .48^a^ | .30^a^ | .49^a^ |  |
| Sig (2-tailed) | .22 | .56 | .02 | < .001 | < .001 | < .001 | < .001 |  |
|  |  |  |  |  |  |  |  |  |
| 1. Depressive Symptoms | -.22 ^a^ | -.12 | -.21^a^ | .31^a^ | -.64^a^ | -.32^a^ | -.69^a^ | -.50^a^ |
| Sig (2-tailed) | < .001 | .06 | < .001 | < .001 | < .001 | < .001 | < .001 | < .001 |
|  |  |  |  |  |  |  |  |  |

*Note.* Living Situation = Living with others (anchored against living alone), Gender = Male (anchored against referent Female, Chronic Conditions = Number of chronic health conditions. ^a^Correlation is significant at the .01 level (2-tailed). ^b^Correlation is significant at the .05 level (2-tailed).
