# Supplementary material for: Online-Delivered Group and Personal Exercise Programs to Support Low Active Older Adults’ Mental Health During the COVID-19 Pandemic: Randomized Controlled Trial
Source: J Med Internet Res. 2021 Jul 30;23(7):e30709. doi: 10.2196/30709 (PMC8330630; doi:10.2196/30709)
Supplement: Multimedia Appendix 13 [file jmir_v23i7e30709_app13.docx]

**Multimedia Appendix 13. Correlations among study variables at week 4.**

| Variable | 1 | 2 | 3 | 4 | 5 | 6 | 7 | 8 |
| --- | --- | --- | --- | --- | --- | --- | --- | --- |
| 1. Gender |  |  |  |  |  |  |  |  |
| Sig (2-tailed) |  |  |  |  |  |  |  |  |
|  |  |  |  |  |  |  |  |  |
| 1. Age | .04 |  |  |  |  |  |  |  |
| Sig (2-tailed) | .55 |  |  |  |  |  |  |  |
|  |  |  |  |  |  |  |  |  |
| 1. Living Situation | .16^b^ | -.17^a^ |  |  |  |  |  |  |
| Sig (2-tailed) | .01 | .007 |  |  |  |  |  |  |
|  |  |  |  |  |  |  |  |  |
| 1. Chronic Conditions | -.20^a^ | -.02 | -.009 |  |  |  |  |  |
| Sig (2-tailed) | .002 | .81 | .89 |  |  |  |  |  |
|  |  |  |  |  |  |  |  |  |
| 1. Satisfaction with Life | .25^a^ | .05 | .16^b^ | -.20^a^ |  |  |  |  |
| Sig (2-tailed) | < .001 | .50 | .02 | .005 |  |  |  |  |
|  |  |  |  |  |  |  |  |  |
| 1. Physical Health | .02 | .17^b^ | .07 | -.23^a^ | .43^a^ |  |  |  |
| Sig (2-tailed) | .79 | .02 | .31 | .001 | < .001 |  |  |  |
|  |  |  |  |  |  |  |  |  |
| 1. Mental Health | .25^a^ | .17^b^ | .11 | -.24^a^ | .69^a^ | .51^a^ |  |  |
| Sig (2-tailed) | < .001 | .02 | .13 | < .001 | < .001 | < .001 |  |  |
|  |  |  |  |  |  |  |  |  |
| 1. Flourishing | .17^b^ | .07 | .16^b^ | -.20^a^ | .64^a^ | .34^a^ | .61 ^a^ |  |
| Sig (2-tailed) | .01 | .30 | .02 | .004 | < .001 | < .001 | < .001 |  |
|  |  |  |  |  |  |  |  |  |
| 1. Depressive Symptoms | -.25^a^ | -.04 | -.20^a^ | .28^a^ | -.67^a^ | -.42^a^ | -.71^a^ | -.69^a^ |
| Sig (2-tailed) | < .001 | .62 | .005 | < .001 | < .001 | < .001 | < .001 | < .001 |
|  |  |  |  |  |  |  |  |  |

*Note.* Living Situation = Living with others (anchored against living alone), Gender = Male (anchored against referent Female, Chronic Conditions = Number of chronic health conditions. ^a^Correlation is significant at the .01 level (2-tailed). ^b^Correlation is significant at the .05 level (2-tailed).
