# Supplementary material for: Online-Delivered Group and Personal Exercise Programs to Support Low Active Older Adults’ Mental Health During the COVID-19 Pandemic: Randomized Controlled Trial
Source: J Med Internet Res. 2021 Jul 30;23(7):e30709. doi: 10.2196/30709 (PMC8330630; doi:10.2196/30709)
Supplement: Multimedia Appendix 16 [file jmir_v23i7e30709_app16.docx]

**Multimedia Appendix 16. Correlations among study variables at week 10.**

| Variable | 1 | 2 | 3 | 4 | 5 | 6 | 7 | 8 |
| --- | --- | --- | --- | --- | --- | --- | --- | --- |
| 1. Gender |  |  |  |  |  |  |  |  |
| Sig (2-tailed) |  |  |  |  |  |  |  |  |
|  |  |  |  |  |  |  |  |  |
| 1. Age | .04 |  |  |  |  |  |  |  |
| Sig (2-tailed) | .55 |  |  |  |  |  |  |  |
|  |  |  |  |  |  |  |  |  |
| 1. Living Situation | .16^b^ | -.17^a^ |  |  |  |  |  |  |
| Sig (2-tailed) | .01 | .007 |  |  |  |  |  |  |
|  |  |  |  |  |  |  |  |  |
| 1. Chronic Conditions | -.20^a^ | -.02 | -.009 |  |  |  |  |  |
| Sig (2-tailed) | .002 | .81 | .89 |  |  |  |  |  |
|  |  |  |  |  |  |  |  |  |
| 1. Satisfaction with Life | .19^b^ | .08 | .15^b^ | -.20^a^ |  |  |  |  |
| Sig (2-tailed) | .008 | .29 | .05 | .006 |  |  |  |  |
|  |  |  |  |  |  |  |  |  |
| 1. Physical Health | .05 | .11 | .09 | -.24^a^ | .48^a^ |  |  |  |
| Sig (2-tailed) | .49 | .12 | .20 | .001 | < .001 |  |  |  |
|  |  |  |  |  |  |  |  |  |
| 1. Mental Health | .14 | .09 | .19^a^ | -.18 ^b^ | .74^a^ | .60^a^ |  |  |
| Sig (2-tailed) | .06 | .22 | .009 | .02 | < .001 | < .001 |  |  |
|  |  |  |  |  |  |  |  |  |
| 1. Flourishing | .006 | -.002 | .20^a^ | -.18^b^ | .62^a^ | .44^a^ | .64^a^ |  |
| Sig (2-tailed) | .93 | .98 | .006 | .01 | < .001 | < .001 | < .001 |  |
|  |  |  |  |  |  |  |  |  |
| 1. Depressive Symptoms | -.20 ^a^ | -.009 | -.25 ^a^ | .27^a^ | -.68^a^ | -.51^a^ | -.69^a^ | -.70^a^ |
| Sig (2-tailed) | .006 | .91 | < .001 | < .001 | < .001 | < .001 | < .001 | < .001 |
|  |  |  |  |  |  |  |  |  |

*Note.* Living Situation = Living with others (anchored against living alone), Gender = Male (anchored against referent Female, Chronic Conditions = Number of chronic health conditions. ^a^Correlation is significant at the .01 level (2-tailed). ^b^Correlation is significant at the .05 level (2-tailed).
