# Supplementary material for: Online-Delivered Group and Personal Exercise Programs to Support Low Active Older Adults’ Mental Health During the COVID-19 Pandemic: Randomized Controlled Trial
Source: J Med Internet Res. 2021 Jul 30;23(7):e30709. doi: 10.2196/30709 (PMC8330630; doi:10.2196/30709)
Supplement: Multimedia Appendix 17 [file jmir_v23i7e30709_app17.docx]

**Multimedia Appendix 17. Correlations among study variables at week 12.**

| Variable | 1 | 2 | 3 | 4 | 5 | 6 | 7 | 8 |
| --- | --- | --- | --- | --- | --- | --- | --- | --- |
| 1. Gender |  |  |  |  |  |  |  |  |
| Sig (2-tailed) |  |  |  |  |  |  |  |  |
|  |  |  |  |  |  |  |  |  |
| 1. Age | .04 |  |  |  |  |  |  |  |
| Sig (2-tailed) | .55 |  |  |  |  |  |  |  |
|  |  |  |  |  |  |  |  |  |
| 1. Living Situation | .16^b^ | -.17^a^ |  |  |  |  |  |  |
| Sig (2-tailed) | .01 | .007 |  |  |  |  |  |  |
|  |  |  |  |  |  |  |  |  |
| 1. Chronic Conditions | -.20^a^ | -.02 | -.009 |  |  |  |  |  |
| Sig (2-tailed) | .002 | .81 | .89 |  |  |  |  |  |
|  |  |  |  |  |  |  |  |  |
| 1. Satisfaction with Life | .14^b^ | -.01 | .07 | -.18^b^ |  |  |  |  |
| Sig (2-tailed) | .05 | .86 | .36 | .01 |  |  |  |  |
|  |  |  |  |  |  |  |  |  |
| 1. Physical Health | .03 | .19^b^ | -.01 | -.20^a^ | .44^a^ |  |  |  |
| Sig (2-tailed) | .69 | .01 | .88 | .006 | < .001 |  |  |  |
|  |  |  |  |  |  |  |  |  |
| 1. Mental Health | .14^b^ | .14 | .06 | -.23^b^ | .66^a^ | .53^a^ |  |  |
| Sig (2-tailed) | .05 | .06 | .45 | .002 | < .001 | < .001 |  |  |
|  |  |  |  |  |  |  |  |  |
| 1. Flourishing | .08 | -.02 | .17^b^ | -.22^b^ | .65^a^ | .47^a^ | .58^a^ |  |
| Sig (2-tailed) | .28 | .82 | .02 | .003 | < .001 | < .001 | < .001 |  |
|  |  |  |  |  |  |  |  |  |
| 1. Depressive Symptoms | -.17^b^ | -.02 | -.13 | .33^a^ | -.62^a^ | -.45^a^ | -.68^a^ | -.67^a^ |
| Sig (2-tailed) | .02 | .83 | .07 | < .001 | < .001 | < .001 | < .001 | < .001 |
|  |  |  |  |  |  |  |  |  |

*Note.* Living Situation = Living with others (anchored against living alone), Gender = Male (anchored against referent Female, Chronic Conditions = Number of chronic health conditions. ^a^Correlation is significant at the .01 level (2-tailed). ^b^Correlation is significant at the .05 level (2-tailed).
